# Associations of vitamin D-related single nucleotide polymorphisms with post-stroke depression among ischemic stroke population

**DOI:** 10.3389/fpsyt.2023.1148047

**Published:** 2023-06-02

**Authors:** Dongren Sun, Mingyu Song, Chang Zeng, Hengshu Chen, Jingyuan Zhang, Fan Liu, Shihang Luo, Qiao Liao, Yeqing Xiao, Weiye Xu, Danfeng Zeng, Zheren Tan, Fafa Tian, Xia Huang

**Affiliations:** ^1^Department of Neurology, Xiangya Hospital, Central South University, Changsha, China; ^2^National Clinical Research Center for Geriatric Disorders, Xiangya Hospital, Central South University, Changsha, China; ^3^Health Management Center, Xiangya Hospital, Central South University, Changsha, Hunan, China; ^4^Department of Neurology, Hengyang Central Hospital, Hengyang, Hunan, China; ^5^Department of Human Anatomy and Neurobiology, School of Basic Medicine, Central South University, Changsha, Hunan, China; ^6^Department of Neurology, Xiangtan Central Hospital, Xiangtan, Hunan, China; ^7^Department of Critical Care Medicine, The First People’s Hospital of Huaihua, Huaihua, Hunan, China

**Keywords:** vitamin D, post-stroke depression, *VDR*, *CYP2R1*, *CYP24A1*, *CYP27B1*

## Abstract

**Objective:**

To investigate the relationship between single nucleotide polymorphisms (SNPs) related to vitamin D (VitD) metabolism and post-stroke depression (PSD) in patients with ischemic stroke.

**Methods:**

A total of 210 patients with ischemic stroke were enrolled at the Department of Neurology in Xiangya Hospital, Central South University, from July 2019 to August 2021. SNPs in the VitD metabolic pathway (*VDR*, *CYP2R1*, *CYP24A1*, and *CYP27B1*) were genotyped using the SNPscan^™^ multiplex SNP typing kit. Demographic and clinical data were collected using a standardized questionnaire. Multiple genetic models including dominant, recessive, and over-dominant models were utilized to analyze the associations between SNPs and PSD.

**Results:**

In the dominant, recessive, and over-dominant models, no significant association was observed between the selected SNPs in the *CYP24A1* and *CYP2R1* genes and PSD. However, univariate and multivariate logistic regression analysis revealed that the *CYP27B1* rs10877012 G/G genotype was associated with a decreased risk of PSD (OR: 0.41, 95% CI: 0.18–0.92, *p* = 0.030 and OR: 0.42, 95% CI: 0.18–0.98, *p* = 0.040, respectively). Furthermore, haplotype association analysis indicated that rs11568820-rs1544410-rs2228570-rs7975232-rs731236 CCGAA haplotype in the *VDR* gene was associated with a reduced risk of PSD (OR: 0.14, 95% CI: 0.03–0.65, *p* = 0.010), whereas no significant association was observed between haplotypes in the *CYP2R1* and *CYP24A1* genes and PSD.

**Conclusion:**

Our findings suggest that the polymorphisms of VitD metabolic pathway genes *VDR* and *CYP27B1* may be associated with PSD in patients with ischemic stroke.

## Introduction

Post-stroke depression (PSD) is a prevalent complication of stroke events, affecting approximately one-third of stroke survivors at any given time after a stroke. Individuals with PSD have a poor prognosis, with prolonged hospital stays, impaired neurological recovery, recurrent vascular events, poor quality of life, and increased mortality, leading to serious negative consequences for individuals, families, and society (1, 2). Identifying susceptibility factors for PSD is crucial. Accumulating evidence suggests that PSD development is influenced by various factors, including biological and psychosocial factors (2). Additionally, genetic links have been established between PSD and the apolipoprotein E gene, inflammatory cytokine genes, methylenetetrahydrofolate reductase gene, and brain-derived neurotrophic factor polymorphisms (3–6).

Vitamin D (VitD) is involved in many of the physiological activities of the brain, including the coordination of neurotrophic factors, immune regulation of the nervous system and neuroprotection, among other processes (7). During VitD metabolism, sunlight acts on 7-dehydrocholesterol in the skin epidermis, producing vitamin D3. The liver primarily produces 25 (OH) D through hydroxylation by the hepatic cytochrome P450 enzyme CYP2R1. The second hydroxylation to the active form 1,25 (OH)2D in the kidney is catalyzed by the cytochrome P450 enzyme *CYP27B1* (8, 9). Most of the biological effects of VitD are achieved through VDR-mediated regulation. VitD binds to VDR and acts on target genes to regulate various metabolic processes, cell adhesion, tissue differentiation, development, and angiogenesis (10–12). Several studies have confirmed the association of VitD levels with PSD development. For instance, Gu et al. found that low serum VitD levels were independently associated with PSD (13, 14), consistent with previous studies (15–17). Bahrami et al. also suggested that genetic susceptibility accounts for a considerable proportion of the heterogeneity in VitD levels (8). Hence, genetic variations in the relevant genes may alter enzymatic activity in the VitD metabolic pathway, leading to altered VitD concentrations.

To the best of our knowledge, no studies have examined the association between VitD-related gene polymorphisms and PSD. Given the crucial role that VitD plays in depression pathogenesis, we selected SNP loci that may affect VitD metabolism, especially those involving exonic and 5′-flanking regions. This study aimed to investigate the association between selected SNPs in *VDR* (rs11568820, rs1544410, rs2228570, rs7975232, rs731236), *CYP2R1* (rs12794714, rs10741657, rs7129781), *CYP24A1* (rs2296241, rs2248137, rs2248359, rs2762939, rs2296239, rs2274133), *CYP27B1* (rs10877012), and PSD to identify genetic susceptibility loci for PSD and further explore its pathogenesis.

## Materials and methods

### Participants

We conducted a cross-sectional study at the Department of Neurology in Xiangya Hospital, Central South University from July 2019 to August 2021. The study included patients who had experienced an ischemic stroke. A standardized questionnaire was used to collect patient demographic and clinical characteristics such as age, gender, body mass index (BMI), hypertensive disorders, diabetes, smoking habits, history of alcohol consumption, and stroke location (anterior, posterior or both). Of note, the Mini-Mental State Examination (MMSE) score was used to quantify the cognitive status of the enrolled patients. The Hamilton Depression Scale-17 (HAMD-17) is used to determine a patient’s depressive status. The questionnaire was administered by researchers who received uniform training. BMI was calculated by dividing body weight (in kilograms) by the square of height (in meters). PSD was defined as a HAMD-17 score of >7 at 2 weeks after stroke onset, and the scale’s reliability and validity in Chinese populations have been previously demonstrated (18–20). Inclusion criteria were: (1) patients not older than 80 years; (2) patients with cerebral infarction onset within 14 days; (3) acute ischemic stroke event confirmed by intracranial CT or/and MRI; and (4) subjects able to complete the scale assessment normally. Exclusion criteria were: (1) difficulty in cooperating due to their understanding; (2) presence of other depression-related neurological disorders, including but not limited to Parkinson’s disease, Lewy body dementia, multiple system atrophy; and (3) past diagnosis of depression, bipolar disorder and other psychiatric disorders, regardless of whether the subject is taking anti-anxiety or anti-depressant medication.

This study was approved by the Ethical Investigation and Review Committee of Xiangya Hospital, Central South University, and written informed consent was obtained from all participants or their legal guardians prior to enrollment.

### Single nucleotide polymorphisms selection and genotyping

We have selected 15 SNPs (minor allele frequency > 5%) from the *VDR*, *CYP2R1*, *CYP27B1*, and *CYP24A1* genes or their expression that have previously been reported to be associated with stroke or depression, based on the National Center for Biotechnology Information SNP database^1^ (Supplementary Table S1). After an 8-h fasting period, 3–5 mL of blood was collected from each subject using ethylene diamine tetraacetic acid (EDTA) anticoagulation tubes. The plasma and blood cells were separated by centrifugation at a low temperature (4°C) and stored separately at −80°C until DNA extraction. Genotyping was performed using the high specificity of the ligase ligation reaction to identify alleles at the SNP. This was followed by the introduction of non-specific sequences of different lengths at the end of the ligation probe and by the ligase addition reaction to obtain ligation products of different lengths corresponding to the SNP, PCR amplification of the ligation products using fluorescently labeled universal primers, and electrophoretic separation of the amplified products by fluorescence capillary electrophoresis. Raw data collected on the ABI3730XL sequencer were analyzed using GeneMapper 4.1 software (AppliedBiosystems, USA). The SNPscan^™^ multiplex SNP typing kit was used for genotyping with technical support from the Center for Genetic and Genomic Analysis, Genesky Biotechnologies (Inc., Shanghai, China). This technique has been previously reported in the literature (21, 22). The quality control analysis included a random selection of samples for reproducibility assessment and negative controls. Only individuals with an SNP detection rate of >99.5% were included in the final study.

### Statistical analysis

The statistical analyses were conducted using SPSS version 26.0, R software version 4.2.1 with the gtsummary package version 1.6.2 and pwr package version 1.3.0. Continuous variables were presented as mean ± standard deviation (SD). Hardy–Weinberg equilibrium was assessed using the chi-square test. Categorical variables and genotype frequencies were analyzed using Pearson’s chi-square test or Fisher’s exact test. Continuous variables that followed a normal distribution were analyzed using Student’s *t*-test, and those that did not follow a normal distribution were analyzed using the Mann–Whitney *U* test. The risk assessment was performed using univariate logistic regression models, with the odds ratio (OR) and 95% confidence interval (95% CI) being reported as the outcome measures. Entries with *p*-values at borderline significance in the univariate analysis (*p* < 0.1) were included in the multivariate logistic regression analysis. We investigated the gene-phenotype associations using dominant, recessive, and over-dominant models. The Hardy–Weinberg equilibrium and haplotype analyses were performed using SNPstat software (23). Power calculations for the current study were performed using R software. A power value greater than 0.8 was considered to indicate sufficient statistical power. A *p*-value of less than 0.05 was considered statistically significant. Multiple comparisons were corrected by the simple Bonferroni method (24, 25). The threshold of significance was set at *p* < 0.05/15 = 0.003, with *p* < 0.05 and *p* > 0.003 considered nominally significant. For SNPs that reach significant or nominally significant levels, we use the GWAS4D online tool for functional annotation (26).

## Results

### Clinical features and statistical power

A total of 210 patients in the acute phase of ischemic stroke were ultimately included in this study. Table 1 presents the baseline clinical data between the PSD and non-post-stroke depression (NPSD) groups. Of the participants, 109 (51.9%) were PSD and 101 (48.1%) were NPSD. There were more male patients in the NPSD group (52.4%) compared to the PSD group (47.6%), while more female patients were in the PSD group (61.5%) compared to the NPSD group (38.5%). Although the NPSD group had a higher proportion of smokers and drinkers (53.0 and 51.4%, respectively) and the PSD group had a higher proportion of patients with hypertension and diabetes than the NPSD group (50.7 and 61.9%, respectively), these demographic and clinical characteristics did not differ significantly between the PSD and NPSD groups. Despite no significant difference in stroke location between the two groups, the PSD group had lower MMSE scores compared to the NPSD group (23.82 ± 4.54 vs. 25.46 ± 4.33, *p* = 0.002). The calculated power value using the pwr package of R software was 1, indicating sufficient statistical power in our study.

**Table 1:** Demographic and clinical characteristics of the study participants.

	PSD	NPSD	*P*-value
Age/years	58.08 ± 9.58	56.54 ± 11.96	0.590
Female, n%	40 (61.5%)	25 (38.5%)	0.060
Male, n%	69 (47.6%)	76 (52.4%)	
Body mass index	23.58 ± 2.66	23.82 ± 2.92	0.850
Smoking, n%			
Yes	55 (47.0%)	62 (53.0%)	0.110
No	54 (58.1%)	39 (41.9%)	
Drinking, n%			
Yes	52 (48.6%)	55 (51.4%)	0.330
No	57 (55.3%)	46 (44.7%)	
Hypertension, n%	74 (50.7%)	72 (49.3%)	0.590
Diabetes, n%	39 (61.9%)	24 (38.1%)	0.060
Stroke location			
Anterior	62 (61.4%)	76 (69.7%)	0.400
Both	6 (5.9%)	4 (3.7%)	
Posterior	33 (32.7%)	29 (26.6%)	
MMSE	23.82 ± 4.54	25.46 ± 4.33	0.002

The data are listed as the mean ± SD or numbers of patients when appropriate. Stroke location represents either anterior or posterior circulation or both.

### SNP’s characteristics

All 15 SNPs were found to be in Hardy–Weinberg equilibrium (*p* > 0.05). Table 2 presents the results of the analysis of genotype and allele distribution, dominant, recessive, and over-dominant models for all 15 SNPs. The study found no significant differences in the genotypes and allele frequencies of the selected *VDR*, *CYP24A1*, *CYP2R1*, and *CYP27B1* genes between the PSD and NPSD groups (*p* > 0.05). In addition, no significant differences were observed in the three gene models for the *VDR*, *CYP24A1*, and *CYP2R1* genes between the two groups. No significant differences were found in the dominant and over-dominant models for rs10877012. However, univariate and multivariate logistic regression analyses showed a protective effect of *CYP27B1* rs10877012 G/G compared to rs10877012 T/T-G/T for PSD in the recessive model (OR: 0.41, 95% CI: 0.18–0.92, *p* = 0.030 and OR: 0.42, 95% CI: 0.18–0.98, *p* = 0.040, respectively). The functional annotation analysis performed by GWAS4D revealed that the *CYP27B1* rs10877012 variant is involved in encoding a transcript intron (Supplementary Table S2).

**Table 2:** Genetic models of vitamin D metabolic pathway genes of the study participants.

SNP (Gene)	Analyze model	Genotype	PSD	NPSD	OR (95% CI)	*P*-value	OR (95% CI)*	*P*-value*
rs11568820 (*VDR*)	Alleles	C	116 (53.0%)	112 (55.0%)	1.00	0.646	0.660	
		T	102 (47.0%)	90 (45.0%)	1.09 (0.75–1.61)			1.10 (0.73–1.64)
	Genotypes	C/C	30 (27.5%)	32 (31.7%)	1.00	0.790	1.00	0.910
		C/T	56 (51.4%)	48 (47.5%)	1.24 (0.66–2.34)		1.11 (0.57–2.16)	
		T/T	23 (21.1%)	21 (20.8%)	1.17 (0.54–2.53)		1.20 (0.53–2.69)	
	Dominant	C/C	30 (27.5%)	32 (31.7%)	1.00	0.510	1.00	0.690
		C/T–T/T	79 (72.5%)	69 (68.3%)	1.22 (0.67–2.21)		1.13 (0.61–2.12)	
	Recessive	C/C-C/T	86 (78.9%)	80 (79.2%)	1.00	0.960	1.00	0.740
		T/T	23 (21.1%)	21 (20.8%)	1.02 (0.52–1.98)		1.13 (0.56–2.28)	
	Overdominant	C/C-T/T	53 (48.6%)	53 (52.5%)	1.00	0.580	1.00	0.930
		C/T	56 (51.4%)	48 (47.5%)	1.17 (0.68–2.01)		1.03 (0.57–1.83)	
rs1544410 (*VDR*)	Alleles	C	204 (94.0%)	194 (96.0%)	1.00	0.258	1.00	0.380
		T	14 (6.0%)	8 (4.0%)	1.66 (0.68–4.06)		1.49 (0.61–3.66)	
	Genotypes	C/C	96 (88.1%)	93 (92.1%)	1.00	0.380	1.00	0.330
		C/T	12 (11%)	8 (7.9%)	1.45 (0.57–3.72)		1.19 (0.44–3.18)	
		T/T	1 (0.9%)	0 (0%)	NA (0.00-NA)		NA (0.00-NA)	
	Dominant	C/C	96 (88.1%)	93 (92.1%)	1.00	0.330	1.00	0.530
		C/T–T/T	13 (11.9%)	8 (7.9%)	1.57 (0.62–3.97)		1.36 (0.52–3.58)	
	Recessive	C/C-C/T	108 (99.1%)	101 (100%)	1.00	0.250	1.00	0.150
		T/T	1 (0.9%)	0 (0%)	NA (0.00-NA)		NA (0.00-NA)	
	Overdominant	C/C-T/T	97 (89%)	93 (92.1%)	1.00	0.440	1.00	0.740
		C/T	12 (11%)	8 (7.9%)	1.44 (0.56–3.68)		1.18 (0.44–3.14)	
rs2228570 (*VDR*)	Alleles	A	118 (54.0%)	96 (48.0%)	1.00	0.176	1.00	0.300
		G	100 (46.0%)	106 (52.0%)	0.77 (0.52–1.13)		0.82 (0.56–1.20)	
	Genotypes	A/A	35 (32.1%)	25 (24.8%)	1.00	0.430	1.00	0.580
		A/G	48 (44%)	46 (45.5%)	0.75 (0.39–1.43)		0.78 (0.40–1.55)	
		G/G	26 (23.9%)	30 (29.7%)	0.62 (0.30–1.29)		0.67 (0.31–1.43)	
	Dominant	A/A	35 (32.1%)	25 (24.8%)	1.00	0.240	1.00	0.340
		A/G-G/G	74 (67.9%)	76 (75.2%)	0.70 (0.38–1.27)		0.74 (0.39–1.38)	
	Recessive	A/A-A/G	83 (76.2%)	71 (70.3%)	1.00	0.340	1.00	0.440
		G/G	26 (23.9%)	30 (29.7%)	0.74 (0.40–1.37)		0.78 (0.41–1.47)	
	Overdominant	A/A-G/G	61 (56%)	55 (54.5%)	1.00	0.830	1.00	0.870
		A/G	48 (44%)	46 (45.5%)	0.94 (0.55–1.62)		0.95 (0.54–1.68)	
rs7975232 (*VDR*)	Alleles	C	144 (66.0%)	139 (69.0%)	1.00	0.547	1.00	0.370
		A	74 (34.0%)	63 (31.0%)	1.13 (0.75–1.71)		1.22 (0.79–1.90)	
	Genotypes	C/C	45 (41.3%)	49 (48.5%)	1.00	0.430	1.00	0.240
		C/A	54 (49.5%)	41 (40.6%)	1.43 (0.81–2.54)		1.65 (0.90–3.01)	
		A/A	10 (9.2%)	11 (10.9%)	0.99 (0.38–2.55)		1.04 (0.38–2.86)	
	Dominant	C/C	45 (41.3%)	49 (48.5%)	1.00	0.290	1.00	0.150
		C/A-A/A	64 (58.7%)	52 (51.5%)	1.34 (0.78–2.31)		1.52 (0.86–2.71)	
	Recessive	C/C-C/A	99 (90.8%)	90 (89.1%)	1.00	0.680	1.00	0.650
		A/A	10 (9.2%)	11 (10.9%)	0.83 (0.34–2.04)		0.80 (0.31–2.09)	
	Overdominant	C/C-A/A	55 (50.5%)	60 (59.4%)	1.00	0.190	1.00	0.090
		C/A	54 (49.5%)	41 (40.6%)	1.44 (0.83–2.48)		1.64 (0.92–2.91)	
rs731236 (*VDR*)	Alleles	A	205 (94.0%)	191 (95.0%)	1.00	0.819	1.00	0.780
		G	13 (6.0%)	11 (5.0%)	1.10 (0.48–2.52)		1.13 (0.48–2.65)	
	Genotypes	A/A	97 (89%)	90 (89.1%)	1.00	0.510	1.00	0.340
		A/G	11 (10.1%)	11 (10.9%)	0.93 (0.38–2.25)		0.88 (0.34–2.23)	
		G/G	1 (0.9%)	0 (0%)	NA (0.00-NA)		NA (0.00-NA)	
	Dominant	A/A	97 (89%)	90 (89.1%)	1.00	0.980	1.00	1.000
		A/G-G/G	12 (11%)	11 (10.9%)	1.01 (0.43–2.41)		1.00 (0.40–2.49)	
	Recessive	A/A-A/G	108 (99.1%)	101 (100%)	1.00	0.250	1.00	0.150
		G/G	1 (0.9%)	0 (0%)	NA (0.00-NA)		NA (0.00-NA)	
	Overdominant	A/A-G/G	98 (89.9%)	90 (89.1%)	1.00	0.850	1.00	0.760
		A/G	11 (10.1%)	11 (10.9%)	0.92 (0.38–2.22)		0.86 (0.34–2.19)	
rs12794714 (*CYP2R1*)	Alleles	G	135 (62.0%)	131 (65.0%)	1.00	0.534	1.00	0.570
		A	83 (38.0%)	71 (35.0%)	1.13 (0.76–1.69)		1.13 (0.74–1.71)	
	Genotypes	G/G	41 (37.6%)	43 (42.6%)	1.00	0.760	1.00	0.830
		G/A	53 (48.6%)	45 (44.5%)	1.24 (0.69–2.21)		1.19 (0.64–2.19)	
		A/A	15 (13.8%)	13 (12.9%)	1.21 (0.51–2.85)		1.22 (0.50–3.00)	
	Dominant	G/G	41 (37.6%)	43 (42.6%)	1.00	0.460	1.00	0.550
		G/A-A/A	68 (62.4%)	58 (57.4%)	1.23 (0.71–2.14)		1.20 (0.67–2.13)	
	Recessive	G/G-G/A	94 (86.2%)	88 (87.1%)	1.00	0.850	1.00	0.800
		A/A	15 (13.8%)	13 (12.9%)	1.08 (0.49–2.40)		1.11 (0.48–2.57)	
	Overdominant	G/G-A/A	56 (51.4%)	56 (55.5%)	1.00	0.550	1.00	0.670
		G/A	53 (48.6%)	45 (44.5%)	1.18 (0.68–2.03)		1.13 (0.64–2.00)	
rs10741657 (*CYP2R1*)	Alleles	G	153 (70.0%)	139 (69.0%)	1.00	0.760	1.00	0.550
		A	65 (30.0%)	63 (31.0%)	0.94 (0.62–1.42)		0.88 (0.58–1.34)	
	Genotypes	G/G	54 (49.5%)	49 (48.5%)	1.00	0.920	1.00	0.710
		A/G	45 (41.3%)	41 (40.6%)	1.00 (0.56–1.77)		1.00 (0.55–1.82)	
		A/A	10 (9.2%)	11 (10.9%)	0.82 (0.32–2.11)		0.67 (0.26–1.77)	
	Dominant	G/G	54 (49.5%)	49 (48.5%)	1.00	0.880	1.00	0.770
		A/G-A/A	55 (50.5%)	52 (51.5%)	0.96 (0.56–1.65)		0.92 (0.52–1.62)	
	Recessive	G/G-A/G	99 (90.8%)	90 (89.1%)	1.00	0.680	1.00	0.410
		A/A	10 (9.2%)	11 (10.9%)	0.83 (0.34–2.04)		0.67 (0.27–1.71)	
	Overdominant	G/G-A/A	64 (58.7%)	60 (59.4%)	1.00	0.920	1.00	0.830
		A/G	45 (41.3%)	41 (40.6%)	1.03 (0.59–1.78)		1.07 (0.60–1.90)	
rs7129781 (*CYP2R1*)	Alleles	T	187 (86.0%)	166 (82.0%)	1.00	0.314	1.00	0.520
		C	31 (14.0%)	36 (18.0%)	0.76 (0.45–1.29)		0.84 (0.48–1.44)	
	Genotypes	T/T	80 (73.4%)	69 (68.3%)	1.00	0.550	1.00	0.760
		T/C	27 (24.8%)	28 (27.7%)	0.83 (0.45–1.54)		0.89 (0.46–1.70)	
		C/C	2 (1.8%)	4 (4%)	0.43 (0.08–2.43)		0.55 (0.09–3.22)	
	Dominant	T/T	80 (73.4%)	69 (68.3%)	1.00	0.420	1.00	0.610
		T/C-C/C	29 (26.6%)	32 (31.7%)	0.78 (0.43–1.42)		0.85 (0.45–1.59)	
	Recessive	T/T–T/C	107 (98.2%)	97 (96%)	1.00	0.350	1.00	0.520
		C/C	2 (1.8%)	4 (4%)	0.45 (0.08–2.53)		0.57 (0.10–3.30)	
	Overdominant	T/T-C/C	82 (75.2%)	73 (72.3%)	1.00	0.630	1.00	0.780
		T/C	27 (24.8%)	28 (27.7%)	0.86 (0.46–1.59)		0.91 (0.48–1.74)	
rs2296241 (*CYP24A1*)	Alleles	G	127 (58.0%)	125 (62.0%)	1.00	0.449	1.00	0.280
		A	91 (42.0%)	77 (38.0%)	1.16 (0.79–1.72)		1.26 (0.82–1.93)	
	Genotypes	G/G	38 (34.9%)	35 (34.6%)	1.00	0.270	1.00	0.420
		G/A	51 (46.8%)	55 (54.5%)	0.85 (0.47–1.55)		1.05 (0.56–1.97)	
		A/A	20 (18.4%)	11 (10.9%)	1.67 (0.70–3.99)		1.76 (0.72–4.31)	
	Dominant	G/G	38 (34.9%)	35 (34.6%)	1.00	0.970	1.00	0.590
		G/A-A/A	71 (65.1%)	66 (65.3%)	0.99 (0.56–1.75)		1.18 (0.65–2.16)	
	Recessive	G/G-G/A	89 (81.7%)	90 (89.1%)	1.00	0.130	1.00	0.190
		A/A	20 (18.4%)	11 (10.9%)	1.84 (0.83–4.06)		1.71 (0.76–3.86)	
	Overdominant	G/G-A/A	58 (53.2%)	46 (45.5%)	1.00	0.270	1.00	0.670
		G/A	51 (46.8%)	55 (54.5%)	0.74 (0.43–1.27)		0.88 (0.50–1.56)	
rs2248137 (*CYP24A1*)	Alleles	C	129 (59.0%)	121 (60.0%)	1.00	0.880	1.00	0.720
		G	89 (41.0%)	81 (40.0%)	1.03 (0.70–1.52)		1.08 (0.71–1.64)	
	Genotypes	C/C	39 (35.8%)	32 (31.7%)	1.00	0.310	1.00	0.550
		C/G	51 (46.8%)	57 (56.4%)	0.73 (0.40–1.34)		0.84 (0.45–1.58)	
		G/G	19 (17.4%)	12 (11.9%)	1.30 (0.55–3.07)		1.33 (0.55–3.22)	
	Dominant	C/C	39 (35.8%)	32 (31.7%)	1.00	0.530	1.00	0.820
		C/G-G/G	70 (64.2%)	69 (68.3%)	0.83 (0.47–1.48)		0.93 (0.51–1.70)	
	Recessive	C/C-C/G	90 (82.6%)	89 (88.1%)	1.00	0.260	1.00	0.340
		G/G	19 (17.4%)	12 (11.9%)	1.57 (0.72–3.42)		1.47 (0.66–3.28)	
	Overdominant	C/C-G/G	58 (53.2%)	44 (43.6%)	1.00	0.160	1.00	0.370
		C/G	51 (46.8%)	57 (56.4%)	0.68 (0.39–1.17)		0.77 (0.44–1.36)	
rs2248359 (*CYP24A1*)	Alleles	C	142 (65.0%)	131 (65.0%)	1.00	0.951	1.00	0.960
		T	76 (35.0%)	71 (35.0%)	0.99 (0.66–1.48)		1.01 (0.66–1.55)	
	Genotypes	C/C	45 (41.3%)	41 (40.6%)	1.00	0.990	1.00	0.920
		C/T	52 (47.7%)	49 (48.5%)	0.97 (0.54–1.72)		1.10 (0.60–2.02)	
		T/T	12 (11%)	11 (10.9%)	0.99 (0.40–2.50)		0.94 (0.37–2.42)	
	Dominant	C/C	45 (41.3%)	41 (40.6%)	1.00	0.920	1.00	0.820
		C/T–T/T	64 (58.7%)	60 (59.4%)	0.97 (0.56–1.69)		1.07 (0.60–1.90)	
	Recessive	C/C-C/T	97 (89%)	90 (89.1%)	1.00	0.980	1.00	0.800
		T/T	12 (11%)	11 (10.9%)	1.01 (0.43–2.41)		0.89 (0.37–2.17)	
	Overdominant	C/C-T/T	57 (52.3%)	52 (51.5%)	1.00	0.910	1.00	0.700
		C/T	52 (47.7%)	49 (48.5%)	0.97 (0.56–1.66)		1.12 (0.63–1.97)	
rs2762939 (*CYP24A1*)	Alleles	G	165 (76.0%)	149 (74.0%)	1.00	0.650	1.00	0.960
		C	53 (24.0%)	53 (26.0%)	0.90 (0.58–1.40)		0.99 (0.61–1.60)	
	Genotypes	G/G	61 (56%)	53 (52.5%)	1.00	0.880	1.00	0.970
		G/C	43 (39.5%)	43 (42.6%)	0.87 (0.50–1.52)		0.95 (0.53–1.70)	
		C/C	5 (4.6%)	5 (5%)	0.87 (0.24–3.17)		1.10 (0.29–4.19)	
	Dominant	G/G	61 (56%)	53 (52.5%)	1.00	0.610	1.00	0.890
		G/C-C/C	48 (44%)	48 (47.5%)	0.87 (0.50–1.50)		0.96 (0.55–1.69)	
	Recessive	G/G-G/C	104 (95.4%)	96 (95%)	1.00	0.900	1.00	0.860
		C/C	5 (4.6%)	5 (5%)	0.92 (0.26–3.29)		1.13 (0.30–4.18)	
	Overdominant	G/G-C/C	66 (60.5%)	58 (57.4%)	1.00	0.650	1.00	0.830
		G/C	43 (39.5%)	43 (42.6%)	0.88 (0.51–1.52)		0.94 (0.53–1.66)	
rs2296239 (*CYP24A1*)	Alleles	T	126 (58.0%)	121 (60.0%)	1.00	0.662	1.00	0.520
		C	92 (42.0%)	81 (40.0%)	1.09 (0.74–1.61)		1.14 (0.76–1.71)	
	Genotypes	T/T	35 (32.1%)	39 (38.6%)	1.00	0.440	1.00	0.210
		C/T	56 (51.4%)	43 (42.6%)	1.45 (0.79–2.66)		1.72 (0.91–3.26)	
		C/C	18 (16.5%)	19 (18.8%)	1.06 (0.48–2.33)		1.10 (0.48–2.53)	
	Dominant	T/T	35 (32.1%)	39 (38.6%)	1.00	0.320	1.00	0.160
		C/T-C/C	74 (67.9%)	62 (61.4%)	1.33 (0.75–2.35)		1.52 (0.84–2.77)	
	Recessive	T/T-C/T	91 (83.5%)	82 (81.2%)	1.00	0.660	1.00	0.580
		C/C	18 (16.5%)	19 (18.8%)	0.85 (0.42–1.74)		0.81 (0.39–1.70)	
	Overdominant	T/T-C/C	53 (48.6%)	58 (57.4%)	1.00	0.200	1.00	0.079
		C/T	56 (51.4%)	43 (42.6%)	1.43 (0.83–2.46)		1.67 (0.94–2.96)	
rs2274133 (*CYP24A1*)	Alleles	A	187 (86.0%)	175 (87.0%)	1.00	0.800	1.00	0.740
		G	31 (14.0%)	27 (13.0%)	1.07 (0.62–1.87)		1.11 (0.60–2.06)	
	Genotypes	A/A	79 (72.5%)	74 (73.3%)	1.00	0.520	1.00	0.410
		A/G	29 (26.6%)	27 (26.7%)	1.01 (0.55–1.86)		1.00 (0.53–1.90)	
		G/G	1 (0.9%)	0 (0%)	NA (0.00-NA)		NA (0.00-NA)	
	Dominant	A/A	79 (72.5%)	74 (73.3%)	1.00	0.900	1.00	0.880
		A/G-G/G	30 (27.5%)	27 (26.7%)	1.04 (0.57–1.91)		1.05 (0.56–1.99)	
	Recessive	A/A-A/G	108 (99.1%)	101 (100%)	1.00	0.250	1.00	0.180
		G/G	1 (0.9%)	0 (0%)	NA (0.00-NA)		NA (0.00-NA)	
	Overdominant	A/A-G/G	80 (73.4%)	74 (73.3%)	1.00	0.980	1.00	0.970
		A/G	29 (26.6%)	27 (26.7%)	0.99 (0.54–1.83)		0.99 (0.52–1.88)	
rs10877012 (*CYP27B1*)	Alleles	T	145 (67.0%)	123 (61.0%)	1.00	0.231	1.00	0.230
		G	73 (33.0%)	79 (39.0%)	0.78 (0.53–1.17)		0.78 (0.52–1.17)	
	Genotypes	T/T	46 (42.2%)	42 (41.6%)	1.00	0.067	1.00	0.110
		G/T	53 (48.6%)	39 (38.6%)	1.24 (0.69–2.23)		1.16 (0.63–2.15)	
		G/G	10 (9.2%)	20 (19.8%)	0.46 (0.19–1.09)		0.45 (0.18–1.12)	
	Dominant	T/T	46 (42.2%)	42 (41.6%)	1.00	0.930	1.00	0.790
		G/T-G/G	63 (57.8%)	59 (58.4%)	0.97 (0.56–1.69)		0.93 (0.52–1.64)	
	Recessive	T/T-G/T	99 (90.8%)	81 (80.2%)	1.00	**0.027**	1.00	**0.040**
		G/G	10 (9.2%)	20 (19.8%)	**0.41 (0.18–0.92)**		**0.42 (0.18–0.98)**	
	Overdominant	T/T-G/G	56 (51.4%)	62 (61.4%)	1.00	0.140	1.00	0.230
		G/T	53 (48.6%)	39 (38.6%)	1.50 (0.87–2.61)		1.41 (0.80–2.50)	

95% CI, 95% confidence interval; OR, odds-ratio. *+*-value < 0.003 was statistically significant. *P* < 0.05 and *P* > 0.003 was considered nominally significant. *Adjusted model: Adjusted for gender, diabetes and MMSE. MMSE: the Mini-Mental State Examination. Bolded means the value is significant.

### Haplotype association analysis

In the Supplementary materials, we presented the results of the linkage disequilibrium analysis in Supplementary Table S3 and Supplementary Figures S1, S2. The haplotype analysis results were provided in Table 3 and Supplementary Tables S4, S5. Our analysis indicates that the haplotypes of *CYP2R1* and *CYP24A1* genes are not significantly associated with PSD. However, we observed a significant association between the *VDR* rs11568820-rs1544410-rs2228570-rs7975232-rs731236 CCGAA haplotype and a reduced risk of PSD (OR: 0.14, 95% CI: 0.03–0.65, *p* = 0.010).

**Table 3:** Haplotype analysis result of *VDR.*

*VDR*	rs11568820	rs1544410	rs2228570	rs7975232	rs731236	Freq	OR (95% CI)	*P*-value
1	C	C	A	C	A	0.228	1	–
2	T	C	A	C	A	0.1591	0.85 (0.35–2.03)	0.710
3	C	C	G	C	A	0.1568	0.92 (0.39–2.13)	0.840
4	T	C	G	C	A	0.1299	0.54 (0.24–1.24)	0.150
5	T	C	G	A	A	0.083	3.71 (0.90–15.24)	0.070
6	C	C	G	A	A	0.0751	**0.14 (0.03–0.65)**	**0.013**
7	T	C	A	A	A	0.0658	0.73 (0.21–2.53)	0.610
8	C	C	A	A	A	0.0403	6.18 (0.71–53.79)	0.100
9	C	T	G	A	G	0.0218	0.80 (0.18–3.60)	0.770
10	T	T	G	A	G	0.0157	1.31 (0.11–15.05)	0.830
Rare	*	*	*	*	*	0.0245	2.54 (0.53–12.19)	0.250

Freq: haplotype frequency. Bolded means the value is significant. *Reference to rare haplotypes as there is no symbol to identify the group.

## Discussion

To the best of our knowledge, our study was the first to investigate the potential association between VitD-related gene polymorphisms and PSD in the Chinese population with acute ischemic stroke. We conducted multiple genetic models to analyze the selected genetic polymorphisms of *CYP24A1*, *CYP2R1*, and PSD, but did not observe any significant association. However, we observed that the *CYP27B1* rs10877012 G/G genotype was associated with a reduced risk of PSD. Furthermore, we found that the rs11568820-rs1544410-rs2228570-rs7975232-rs731236 CCGAA haplotype of the *VDR* gene was associated with a reduced risk of PSD, indicating an 0.86-fold protective effect against PSD. These findings provide important insights into the potential genetic risk factors for PSD in stroke patients.

Numerous studies have investigated the association between five SNPs in the *VDR* gene and depression. For instance, Kuningas et al. demonstrated that the *VDR* BsmI (C/T) and TaqI (A/G) genotypes could influence cognitive function and depressive symptoms (27). Similarly, other researchers have explored the role of TaqI, ApaI, and BsmI polymorphisms of the *VDR* gene in different contexts. A study analyzing 748 study participants found that TaqI C/C or C/T genotype was associated with emotional response and that patients with autoimmune hepatitis carrying TaqI C/C, BsmI A/A, and ApaI A/A had worse physical, social, emotional, and psychological functioning (28). Another study of survival and health effects in people over 90 years old showed that carriers of *VDR* rs2228570 A/A and/or G/A had little effect on longevity but may affect a variety of pathophysiologically relevant functions, including a significantly lower prevalence of depression (29). However, some researchers have reached opposing conclusions. For example, Groot et al. investigated the association between VitD levels, selected VitD synthesis-related genes, and depressive symptoms. They found that low serum 25 (OH) D levels were associated with higher depressive symptom scores, but no association was found between selected VitD loci and depression (30). A matched case–control study conducted by Lye et al. in Kuala Lumpur and Selangor included 600 participants genotyping three adjacent SNPs (BsmI, ApaI, and TaqI) of the *VDR* gene. They found no statistical association between the nine genotypes of BsmI, ApaI, and TaqI and the risk of developing major depression, but the BsmI-ApaI-TaqI TAC (BAt) haplotype of the *VDR* gene increased susceptibility to major depression (31). Our study also investigated the association between *VDR* gene polymorphisms and PSD in a Chinese acute ischemic stroke population. Interestingly, we found that the CCGAA haplotype of the *VDR* gene was associated with a reduced risk of PSD. It is important to note that the study by Lye et al. was conducted in a major depression population, which is notably inconsistent with a stroke population.

To the best of our knowledge, there have been no studies investigating the relationship between the *CYP2R1* gene and depression in the context of acute ischaemic stroke. However, a meta-analysis conducted in 2018 reported that the rs10741657 A/A genotype of the *CYP2R1* gene influences 25 (OH) D levels and is associated with VitD deficiency (32). Previous genome-wide association studies have identified a robust association between *CYP2R1* A/G and *CYP24A1* T/C genotypes and VitD levels (33). Adalı et al. found that genotype frequencies and polymorphic allele frequencies of *CYP24A1* rs927650 and *CYP2R1* rs10741657 were not associated with ischaemic stroke risk in the Turkish population (34). It is important to note that our study did not find an association between the genotype and allele frequencies of *CYP2R1* rs7129781, rs10741657, and rs12794714 and PSD in the Chinese acute ischaemic stroke population. However, it should be acknowledged that the association between *CYP2R1* and PSD in patients with onset beyond 2 weeks still requires further clarification, as our study did not conduct longer follow-up assessments.

*CYP24A1* is an important regulator of blood circulation and intracellular VitD levels, playing a critical role in activating and regulating various cellular pathways (10). The *CYP24A1* rs1570669 A/G genotype reduced susceptibility to ischemic stroke in both female and male patients, while the rs2296241 A/G genotype reduced risk only in male patients. Conversely, the rs6068816 T/C and rs2762934 A/G genotypes were associated with an increased risk of ischemic stroke (35). Rats exhibiting depression-like symptoms demonstrated increased expression of *CYP27B1*, *CYP24A1* and VDR in the hippocampus and elevated levels of 1,25 (OH)2D (36). However, another animal study showed no change in *CYP27B1* and *CYP24A1* expression in the kidneys of depressed rats (37). In a post-mortem study of suicide conducted by Postolache et al. increased expression of the *VDR* gene and regular expression of the *CYP27B1* or *CYP24A1* genes were observed in the brains of depressed patients (38). Unfortunately, our study did not reveal any statistical association between the selected *CYP24A1* locus and the development of PSD, suggesting that this relationship may need to be explored in future studies with larger sample populations.

*CYP27B1*, which is essential for the VitD metabolic pathway, serves as the 1-α-hydroxylase in humans (39). Although no studies have investigated the relationship between *CYP27B1* and PSD, animal studies have revealed that increased *CYP27B1* expression is associated with depression (36). Moreover, previous research has demonstrated that inactivation or deletion of *CYP27B1* due to mutations causes VitD-dependent rickets type 1 (10, 40). Fam et al. reported that the *CYP24A1* rs2762939 G/G genotype was significantly associated with stroke-like vascular acute events, also known as acute coronary syndrome, while the genotypes of *CYP24A1* rs4809960 and *CYP27B1* rs703842 were not associated with the incidence of acute coronary syndrome (41). In our study, we found a protective effect of the *CYP27B1* rs10877012 G/G genotype compared to the rs10877012 T/T-G/T genotypes in relation to PSD (OR: 0.41, 95% CI: 0.18–0.92, *p* = 0.030), indicating that genes involved in the VitD metabolic pathway may play a role in the development of PSD. Although the functional annotation analysis suggests a potential association of the *CYP27B1* rs10877012 variant with encoding a transcript intron, the underlying mechanisms of its impact on PSD remain unknown. Further investigations using animal models will be necessary to explore the current findings.

In addition, an authoritative meta-analysis showed that the prevalence of early-onset PSD seems to be higher than the one-third reported in most studies, at around 48% (42), which is consistent with our study findings. We used HAMD-17 to identify PSD, while some previous PSD studies have used DSM-5, the nine-item Patient Health Questionnaire, the Center of Epidemiological Studies-Depression Scale, and the Hospital Anxiety and Depression Scale (43–45). Although these tools have been proven to be useful in stroke and are suitable for interviewers to manage depression symptoms discovery or screening tools in busy clinical environments, the heterogeneity between different scales may have led to inconsistent prevalence rates of post-stroke depression.

Our study provides important insights into the genetic pathogenesis of PSD by exploring the association between VitD-related genes and PSD. However, there are several limitations that need to be acknowledged. Firstly, the current study is limited by a small sample size, which is partly due to the heavy clinical workload during the COVID-19 pandemic and China’s zero-clearance policy, which restricted the enrollment of additional eligible patients. Our research, as a preliminary discovery, needs to be validated in a larger sample size to confirm the reliability of our findings. Secondly, our study was conducted primarily in the Chinese Han population, and ethnic differences in PSD may limit the generalizability of our results. Research on other populations in the future remains necessary. Finally, the occurrence of PSD is influenced by multiple factors, including environmental factors, which were not comprehensively assessed in our study. Therefore, further investigations are needed to examine the role of environmental factors and their interactions with genetic factors in the development of PSD.

In conclusion, our study suggests that the *CYP27B1* rs10877012 G/G genotype may act as a genetic protective factor for PSD, while no association was found between the selected *CYP24A1* and *CYP2R1* gene polymorphisms and PSD. Moreover, the *VDR* rs11568820-rs1544410-rs2228570-rs7975232-rs731236 CCGAA haplotype was found to be associated with a reduced risk of PSD. These findings indicate that the polymorphisms of VitD metabolic pathway genes *VDR* and *CYP27B1* may be involved in the development of PSD in the Chinese ischemic stroke population. Further research is needed to verify these findings.

## Data availability statement

The original contributions presented in the study are included in the article/Supplementary material. Further inquiries can be directed to the corresponding authors.

## Ethics statement

The studies involving human participants were reviewed and approved by the Ethical Investigation and Review Committee of Xiangya Hospital, Central South University. The patients/participants provided their written informed consent to participate in this study.

## Author contributions

FT and XH conceived, designed the overall research, and provided important comments on the revision of the article. DS wrote the first manuscript. All authors were involved in the collection, monitoring of the data, and revision of the article, and reviewed and agreed to the submitted version.

## Funding

This work was supported by the China International Medical Foundation (Z-2016-20-2101-03), the National Key Research and Development Program of China (No. 2017YFC1310003), and the Natural Science Foundation of Hunan Province, China (2021JJ41018).

## Conflict of interest

The authors declare that the research was conducted in the absence of any commercial or financial relationships that could be construed as a potential conflict of interest.

## Publisher’s note

All claims expressed in this article are solely those of the authors and do not necessarily represent those of their affiliated organizations, or those of the publisher, the editors and the reviewers. Any product that may be evaluated in this article, or claim that may be made by its manufacturer, is not guaranteed or endorsed by the publisher.

## Footnotes

^1^ http://www.ncbi.nlm.nih.gov/SNP
