# Supplementary material for: Associations of vitamin D-related single nucleotide polymorphisms with post-stroke depression among ischemic stroke population
Source: Front Psychiatry. 2023 Jun 2;14:1148047. doi: 10.3389/fpsyt.2023.1148047 (PMC10317012; doi:10.3389/fpsyt.2023.1148047)
Supplement: Supplementary file 6 [file Data_Sheet_1.PDF]

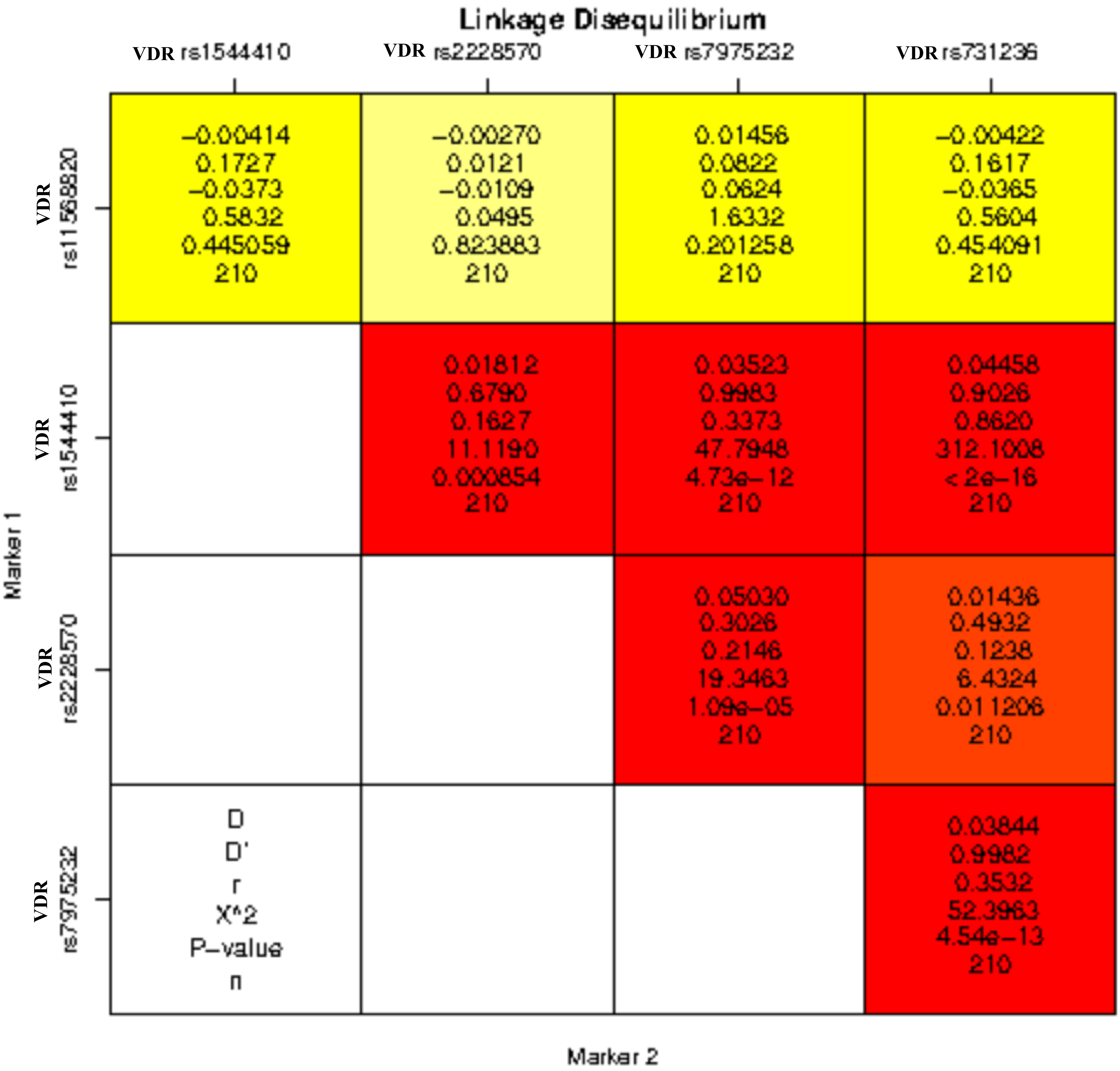
Linkage Disequilibrium
VDR rs1544410
VDR rs2228570
VDR rs7975232
VDR rs731236
VDR rs11568820
VDR rs1544410
VDR rs2228570
VDR rs7975232
Marker 1
Marker 2
-0.00414
0.1727
-0.0373
0.5832
0.445059
210
-0.00270
0.0121
-0.0109
0.0495
0.823883
210
0.01456
0.0822
0.0624
1.6332
0.201258
210
-0.00422
0.1617
-0.0365
0.5604
0.454091
210
0.01812
0.6790
0.1627
11.1190
0.000854
210
0.03523
0.9983
0.3373
47.7948
4.73e-12
210
0.04458
0.9026
0.8620
312.1008
< 2e-16
210
0.05030
0.3026
0.2146
19.3463
1.09e-05
210
0.01436
0.4932
0.1238
6.4324
0.011206
210
D
D'
r
X^2
P-value
n
0.03844
0.9982
0.3532
52.3963
4.54e-13
210
