# Supplementary material for: Associations of vitamin D-related single nucleotide polymorphisms with post-stroke depression among ischemic stroke population
Source: Front Psychiatry. 2023 Jun 2;14:1148047. doi: 10.3389/fpsyt.2023.1148047 (PMC10317012; doi:10.3389/fpsyt.2023.1148047)
Supplement: Supplementary file 7 [file Data_Sheet_2.PDF]

Linkage Disequilibrium

Marker 1 (rows) × Marker 2 (columns)

Marker 1 \ Marker 2	CYP24A1 rs2248137	CYP24A1 rs2248359	CYP24A1 rs2762939	CYP24A1 rs2296239	CYP24A1 rs2274133
CYP24A1 rs2296241	0.20845<br>0.8755<br>0.8668<br>315.597<br>< 2e 16<br>210	0.15941<br>0.7591<br>0.6822<br>196.470<br>< 2e 16<br>210	0.07996<br>0.5280<br>0.3758<br>59.299<br>1.35e 14<br>210	0.00468<br>0.0199<br>0.0194<br>0.158<br>0.691<br>210	0.07932<br>0.9673<br>0.4693<br>92.507<br>< 2e 16<br>210
CYP24A1 rs2248137		0.19026<br>0.9133<br>0.8127<br>277.381<br>< 2e 16<br>210	0.07783<br>0.5181<br>0.3651<br>55.972<br>7.35e 14<br>210	0.00868<br>0.0621<br>0.0359<br>0.542<br>0.462<br>210	0.07875<br>0.9680<br>0.4650<br>90.822<br>< 2e 16<br>210
CYP24A1 rs2248359			0.10262<br>0.6256<br>0.4953<br>103.038<br>< 2e 16<br>210	0.00889<br>0.0617<br>0.0379<br>0.602<br>0.438<br>210	0.08970<br>0.9993<br>0.5451<br>124.804<br>< 2e 16<br>210
CYP24A1 rs2762939				0.00479<br>0.0322<br>0.0224<br>0.210<br>0.646<br>210	0.07756<br>0.7512<br>0.5175<br>112.491<br>< 2e 16<br>210
CYP24A1 rs2296239	D<br>D<br>r<br>X^2<br>P value<br>n				0.01255<br>0.1545<br>0.0739<br>2.296<br>0.130<br>210

Marker 2
